# Flagellar Phenotypes Impact on Bacterial Transport and Deposition Behavior in Porous Media: Case of *Salmonella enterica* Serovar Typhimurium

**DOI:** 10.3390/ijms232214460

**Published:** 2022-11-21

**Authors:** Xin Zheng, Hongjuan Bai, Ye Tao, Mounia Achak, Yannick Rossez, Edvina Lamy

**Affiliations:** 1 Enzyme and Cell Engineering, Centre de Recherche Royallieu, Université de Technologie de Compiègne, UPJV, UMR CNRS 7025, CS 60319, 60203 Compiègne, France; 2 UTC/ESCOM, Centre de Recherche Royallieu, Sorbonne Université, Université de Technologie de Compiègne, EA 4297 TIMR, CS 60319, 60203 Compiègne, France; 3 School of Chemistry and Chemical Engineering, Henan University of Technology, Zhengzhou 450001, China; 4 Science Engineer Laboratory for Energy (LabSIPE), National School of Applied Sciences, Chouaïb Doukkali University, El Jadida 24000, Morocco; 5 Chemical and Biochemical Sciences, Green Process Engineering, Mohammed VI Polytechnic University (UM6P), Benguerir 43150, Morocco; 6 CNRS, UMR 8576—UGSF—Unité de Glycobiologie Structurale et Fonctionnelle, Université de Lille, 59655 Lille, France

**Keywords:** bacterial flagella, *Salmonella enterica*, bacterial transport, porous media, groundwater

## Abstract

Bacterial contamination of groundwater has always been an ecological problem worthy of attention. In this study, *Salmonella enterica* serovar Typhimurium with different flagellar phenotypes mainly characterized during host-pathogen interaction were analyzed for their transport and deposition behavior in porous media. Column transport experiments and a modified mobile-immobile model were applicated on different strains with flagellar motility (wild-type) or without motility (Δ*motAB*), without flagella (Δ*flgKL*), methylated and unmethylated flagellin (Δ*fliB*), and different flagella phases (*fliC^ON^*, *fljB^ON^*). Results showed that flagella motility could promote bacterial transport and deposition due to their biological advantages of moving and attaching to surfaces. We also found that the presence of non-motile flagella improved bacterial adhesion according to a higher retention rate of the Δ*motAB* strain compared to the Δ*flgKL* strain. This indicated that bacteria flagella and motility both had promoting effects on bacterial deposition in sandy porous media. Flagella phases influenced the bacterial movement; the *fliC^ON^* strain went faster through the column than the *fljB^ON^* strain. Moreover, flagella methylation was found to favor bacterial transport and deposition. Overall, flagellar modifications affect *Salmonella enterica* serovar Typhimurium transport and deposition behavior in different ways in environmental conditions.

## 1. Introduction

Groundwater is an important part of water resources and plays a vital role in maintaining ecosystems around the world. However, 80% of wastewater is released into the environment without adequate treatment [1]. Wastewater contains many severe multi-pollutant sources, such as bacteria [2], and some of them are problematic to plants, animals, or human health. These microorganisms reach the groundwater through different layers of the subsurface region [3]. In the environment, a pathogenic bacterium like *Salmonella enterica* is widely disseminated, and it is among the most prevalent food-borne bacterial pathogens [4], causing gastroenteritis worldwide [5]. Water plays an important role in the spreading of this organism among plants, animals, or humans [6]. To take measures to reduce bacterial contamination of water, it is particularly important to understand the transport and adhesion mechanism of bacteria in porous media to protect soil and groundwater from contamination.

To achieve these goals, considerable efforts have been made to decipher the impact of bacterial transport in porous media. Thus, diverse parameters have been investigated: physical properties of porous media such as particle shape [7], soil structure [8,9], pore size [10], porosity [11], hydrodynamic and hydraulic properties of porous media such as flow rate [12], saturation degree [13], and chemical and physicochemical properties related to the liquid phase in which bacteria are transported such as pH and ionic strength [14]. Different properties concerning the bacteria-like cell shape [15], hydrophobicity [16], and motility [17] have been considered. Bacterial motility is the ability of the bacteria to move independently and is considered a virulence factor [18]. Flagella are motility appendages rotating by a reversible motor to ensure their movement toward nutrients and away from toxins [19] in many bacteria. Bacteria have two modes during the swimming motions driven by flagella; runs and tumbles. Runs are long straight swimming, and during tumbles, bacteria stop runs and change their orientation quickly [20]. *Escherichia coli* flagella have been described recently to affect transport and deposition; the flagellated bacteria could cause higher deposition onto quartz sand/silica surfaces [21]. Likewise, Haznedaroglu et al. [22] found that several *Salmonella enterica* serovars which were differentially flagellated displayed altered deposition. Flagellated bacteria had more deposition than non-flagellated strains onto quartz. However, the flagellar phenotype was not the only difference between the serovars with distinct genotypes that were employed in this work.

The multiprotein complex forming the flagellar filament contains mainly one globular protein, the flagellin, but many *S. enterica* serovars express one of two flagellins, FliC or FljB, through a phase variation mechanism [23]. FliC-expressing bacteria are more efficient in colonizing intestinal epithelia than FljB-expressing bacteria [24]. These two flagellins can be methylated on their lysine residues [25] by the methylase FliB, which increases flagellin surface hydrophobicity without affecting bacterial motility [16]. In order to synthesize the flagellar filament, many proteins are needed, including the hook-filament junction protein FlgK and the second hook-filament junction FlgL [26]. Likewise, when the genes *motAB* are inactivated, the bacteria are still flagellated but not motile.

Much research has been carried out to understand the impact of bacterial motility on bacterial transport and deposition in porous media. Recently, Bai et al. [10,27,28] performed bacteria transport and deposition experiments at a laboratory column scale to investigate the simultaneous influence of soil physical and cell properties on the mechanisms governing transport and deposition processes. Different species of motile and non-motile bacteria were used for this work. They found that the transport of non-motile species increased with the increasing heterogeneity of the porous media. This transport reduced non-motile bacteria retention in the porous medium by reducing the contact between bacteria and retention sites. Cell motility could allow the bacteria to swim upstream, leading to longer retention and, subsequently, cell deposition in the explored regions. Furthermore, flagella have also been reported to act as an adhesin on abiotic [29] and on biotic surfaces via lipids [30], in both cases with a better affinity on hydrophobic surfaces. So, the flagella could directly impact bacterial transport through porous media [31,32].

Even though bacterial motility has been widely investigated in the existing literature [33,34,35], there is a lack of understanding about the role of flagellar properties on bacterial transport and deposition behavior in porous media. For this purpose, previous works from Bai et al. were extended here by using one bacterial strain in porous media but with different flagellar phenotypes via mutations. Transport and deposition experiments of *Salmonella enterica* serovar Typhimurium (*S. Typhimurium*) mutants (six different flagellar phenotypes: without flagella, motility, and methylation or expressing one type of flagellin) have been performed on a homogenous sandy medium at column scale under saturated flow conditions. The breakthrough curves (BTCs) and retention profiles (RPs) of *S. Typhimurium* were measured and numerically modeled to decipher the flagellar impact on transport and retention mechanisms.

## 2. Results

### 2.1. Flagella and Their Motility Impact Transport and Deposition Behavior of S. Typhimurium Strain in Sandy Porous Media

**Figure 1:**
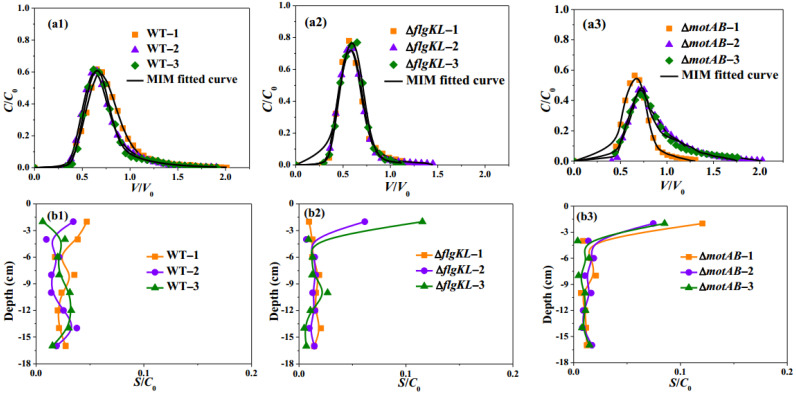
(**a1**–**a3**) Observed (symbols) and fitted (lines) breakthrough curves (BTCs); (**b1**–**b3**) Observed retention profiles (RPs) of *S. Typhimurium* WT motile flagella-on, Δ*flgKL* non-motile flagella-off, and Δ*motAB* non-motile flagella-on mutants. *V* is the volume occupied by water in the column, *V*_0_ is the total pore volume of the column, and *C*, *C*_0_, and *S* are the time-dependent, initial, and solid phase concentration of bacteria respectively.

Several *S. Typhimurium* strains were used to investigate the importance of motile flagella on bacterial transport and deposition in porous media (Table 1): motile wild-type (WT) strain, non-motile with flagella (Δ*motAB*) and non-motile without flagella (Δ*flgKL*). The Δ*motAB* mutant is a non-motile strain without a stator, which is needed for flagella rotation [36]. Bacterial transport and deposition experiments were performed in triplicate, and a good repeatability of breakthrough curves (BTCs), as well as retention profiles (RPs), were obtained for each strain (Figure 1(a1–b3)).

The observed BTCs obtained from WT (Figure 1(a1)) and Δ*motAB* (Figure 1(a3)) exhibited an asymmetrical shape with an obvious tailing, conversely to the BTC symmetrical shape of the strain without flagella (Δ*flgKL*, Figure 1(a2)). However, the flagellated non-motile strain, Δ*motAB*, showed a more obvious tailing compared to WT. Meanwhile, some slight differences were observed between WT and Δ*motAB*: an average higher peak for the WT (before 0.75 *V*/*V*_0_, 0.6 *C*/*C*_0_, Figure 1(a1)) compared to those of Δ*motAB* (around 0.75 *V*/*V*_0_, 0.5 *C*/*C*_0_, Figure 1(a3)). The average mass percentage of Δ*motAB* recovered, M_eff_, was 36.78% (Table 2), lower than WT (47.1%), indicating that bacterial motility could improve bacteria transport through sandy media. This result was consistent with the slightly higher retardation factors observed for Δ*motAB* (average retardation factor, 0.91, Table 2) compared to the WT (0.86, Table 2). The overall bacteria mass recovery (M_total_) was subsequently determined as the sum of M_eff_ and M_retained_. However, different bacteria retention rates in the sand, M_retained_, were obtained for WT (29.7%) and Δ*motAB* (26.5%) strains, and higher M_total_ were obtained for WT (76.8%) in comparison to Δ*motAB* (63.2%) (Table 2) due to the higher recovery of WT strain in the effluent. The observed RPs of Δ*motAB* showed a different behavior compared to WT, especially the bacteria attached to the column inlet (Figure 1(b1,b3)). All replicates of Δ*motAB* showed a non-monotonic distribution profile behavior with a higher number of attached bacteria (*S*/*C*_0_) at the column inlet (layer 0–2 cm). Conversely, the monotonic behavior of WT exhibited no significant difference in retention rate among all layers.

The BTCs of WT and Δ*motAB* were well fitted (Figure 1(a1,a3)) with *R*^2^ > 0.98 (Table 3) using the MIM model. The dispersivity *λ* and mobile water fraction *θ*_m_/*θ* could reflect the flow patterns in porous media; lower *λ* and higher *θ*_m_/*θ* show a more uniform, thus less preferential flow. The dispersivity of WT was 0.49 cm while for Δ*motAB* was 0.38 cm, indicating that WT had more dispersive flow pathways. Slightly lower mobile water fractions *θ*_m_/*θ* were obtained for WT (53%) compared to those of Δ*motAB* (56%) (Table 3), indicating that lower pore water volumes were required for WT transport compared to those of Δ*motAB.* These results showed a different flow pattern between flagellated motile and non-motile strains; the non-motile strain tended to have more homogeneous transport in porous media than the motile strain. The same order of magnitude of the attachment coefficient *k_att_* was obtained for WT and Δ*motAB* on the sandy columns (Table 3). The same tendency was also observed for the detachment coefficient, *k_d_*. The *k_d_* values obtained for both mutants were greater than *k_att_* values (Table 3), indicating that attachment was approaching linear equilibrium sorption conditions.

To examine the importance of flagella presence in transport behavior, two non-motile bacteria were compared: Δ*flgKL* (without flagella) and Δ*motAB* (with flagella). The observed BTCs obtained from Δ*flgKL* occurred earlier than for Δ*motAB* (Figure 1(a2,a3)). Average earlier breakthrough (0.4 *V*/*V*_0_) for Δ*flgKL* was obtained compared to Δ*motAB* (0.5 *V*/*V*_0_). An average higher peak (around 0.75 *C*/*C*_0_) for Δ*flgKL* was observed compared to Δ*motAB* (around 0.6 *C*/*C*_0_). Earlier breakthroughs and higher peaks for Δ*flgKL* were consistent with lower average retardation factors of this strain (0.66, Table 2) compared to Δ*motAB* (0.91).

Similar overall bacteria mass recovery (M_total_) was obtained for both mutants with (63.2% for Δ*motAB*) or without flagella (68.0% for Δ*flgKL*). However, the M_eff_ recovery in the effluent of Δ*flgKL* was higher than for Δ*motAB* (44.7% vs. 36.8%), indicating that the flagella presence may reduce bacterial transport. In addition, Δ*flgKL* exhibited a lower number of bacteria retained in the sand compared to Δ*motAB* (M_retained_ of 23.3% for Δ*flgKL* and of 26.5% for Δ*motAB*, Table 2). The observed RPs of both Δ*flgKL* and Δ*motAB* strains showed similar non-monotonic retention profile behavior (Figure 1b2,b3). Thus, Δ*flgKL* and Δ*motAB* strains showed a higher number of attached bacteria at the column inlet (Figure 1(b2,b3), layer 0–2 cm). This phenomenon indicated that the bacteria were retained mostly in the first layer of the column and displayed the most important step for blocking bacteria contamination.

Δ*flgKL* had lower mean dispersivity *λ* (0.28 cm) but higher mobile water fraction *θ*_m_/*θ* (62%) compared with Δ*motAB* (56%). This phenomenon could be attributed to the difference in flagella presence, meaning that the bacteria with flagella could have a less uniform and more preferential transport in porous media. The mean value of the attachment coefficient *k*_att_ reached 0.06 min^−1^ for Δ*flgKL*, slightly lower than Δ*motAB* (0.09 min^−1^). It showed that Δ*flgKL* is less adhering to sand than Δ*motAB*, a result in agreement with experimental observations. Δ*flgKL* also had a higher detachment coefficient *k*_d_ (0.57 min^−1^) than Δ*motAB* (0.40 min^−1^), indicating a higher possibility of being reversibly detached from the solid grains. 

### 2.2. Flagella Phases Influence Transport and Deposition Behavior of Bacteria in Porous Media

The effect of different flagellins of *S. typhimurium*, here with *fliC^ON^* and *fljB^ON^* strains, was investigated to observe their role in transport and deposition in porous media. We used two mutants that are locked in FliC or FljB, respectively, with different positions and orientations of flagellins. When the flagellum is composed of FliC flagellin, it plays an important role during *Salmonella* infection in mice and humans [37]. 

**Figure 2:**
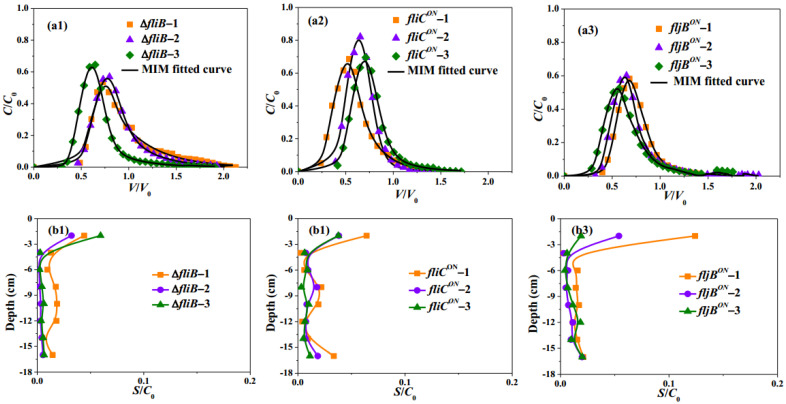
(**a1**–**a3**) Observed (symbols) and fitted (lines) breakthrough curves; (**b1**–**b3**) Observed retention profiles of *S. Typhimurium* motile Δ*fliB*, motile *fliC^ON^*, and motile *fljB^ON^* mutants.

The observed BTCs of *fliC^ON^* and *fljB^ON^* strains (Figure 2(a2,a3)) showed similar arrival breakthroughs for both strains. However, slightly higher average *C*/*C*_0_ peaks were observed for *fliC^ON^* compared to those of *fljB^ON^*. Thus, the average peak *C*/*C*_0_ value of the *fliC^ON^* strain was about 0.7, while the *fljB^ON^* strain was around 0.6. The baseline for *fliC^ON^* and *fljB^ON^* strains was achieved in *V*/*V*_0_ 1.5 and 2.0, respectively. These results showed that *fliC^ON^* goes faster through the column than *fljB^ON^*.

**Figure 3:**
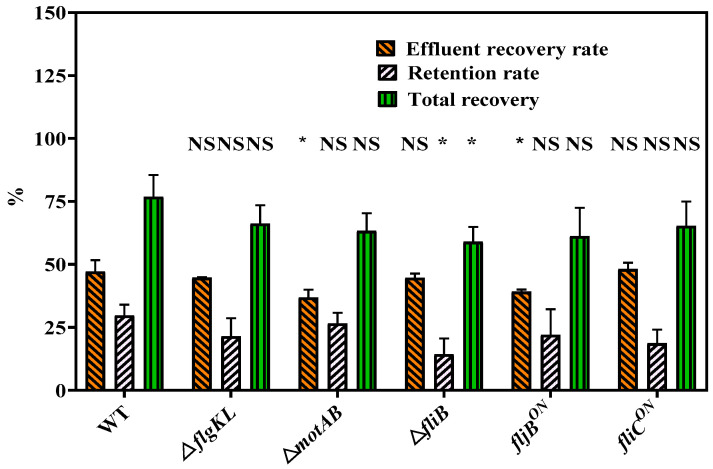
The recovery rate of *S. Typhimurium* different strains. Replicates are shown as mean values, error bars represent standard deviations, and statistical significances were determined by the student’s *t*-test (* = *p* < 0.05; NS = not significant).

The overall bacteria mass recovery (M_total_) reached 61.2% for *fljB^ON^* and 66.6% for the *fliC^ON^* strain. Even though both strains exhibited slightly similar retardation factors (0.78 for *fljB^ON^* and 0.74 *for fliC^ON^*), *the fljB^ON^* strain had a lower percentage of recovery from the effluent than the *fliC^ON^* strain (39.3% vs. 48.1%, respectively, Table 2, Figure 3). In addition, the *fljB^ON^* strain was more retained on the sandy grains (M_retained_ of 21.9% vs. 18.6%, respectively).

Thus, higher recovery in the effluent and lower retention on the sand for the *fliC^ON^* compared to the *fljB^ON^* strain were in agreement with faster transport of the *fliC^ON^* strain observed from BTCs data. Based on the RPs (Figure 2(b2,b3)), the two mutants also showed that the bacteria were more easily attached to the column inlet but with a greater efficacity for *fljB^ON^*. For the *fliC^ON^* and *fljB^ON^* strains, *the fliC^ON^* strain had a little lower dispersivity λ but the same mobile water fraction *θ*_m_/*θ* of 54% as the *fljB^ON^* strain. It seemed that there was no obvious difference between these two strains for the transport pattern. The *fliC^ON^* strain had lower *K_str_* than the *fljB^ON^* strain.

### 2.3. Effects of Flagella Methylation on Transport and Deposition of Bacteria in Porous Media

The posttranslational methylation of flagellin via *fliB* plays an important role in the invasion of host cells [16]. It should be noted that the BTC behavior of the Δ*fliB* strain replicates (Figure 2(a1)), and the average peaks, as well as BTCs arrival, were different from those obtained for WT (Figure 1(a1)). 

The average retardation factor for the Δ*fliB* strain was 0.93 and 0.86 for WT (Table 2), indicating a delay for BTCs of Δ*fliB*. Lower bacteria effluent recovery (M_eff_) was obtained for Δ*fliB* (44.7%) compared to WT (47.1%). Meanwhile, Δ*fliB* presented a much lower retention rate (14.3%) in the sand in comparison to WT (29.7%) (Table 2).

According to the RPs (Figure 1(b1) and Figure 2(b1)), the Δ*fliB* strain was more easily attached to the first layer of the column inlet than WT. The Δ*fliB* strain had lower *λ* (dispersivity of 0.26 cm, Table 3) and higher mobile water fraction *θ*_m_/*θ* (58%) compared to WT (53%), *fliC^ON^* (54%)*,* and *fljB^ON^* strains (54%). Based on these findings, bacteria without flagellin methylation have more uniform and less preferential transport. Δ*fliB* had a higher attachment coefficient *k*_att_ (0.18) than WT (0.09), *fliC^ON^* (0.13), and *fljB^ON^* (0.12), which showed that the Δ*fliB* strain was more likely to be captured by physicochemical mechanisms in the sand than by physical straining. It should be noted that parameters obtained from numerical simulations remained mostly in the same order of magnitude; however, their comparison between various mutants of the same strain allows us to observe some tendencies related to their specific biological properties.

## 3. Discussion

The prokaryotic flagellum is an extraordinary multi-subunit organelle, complex in its regulation and assembly, best known as a motility organelle responsible for bacterial movement, necessary for chemotaxis, and involved in biofilm formation [31,38,39]. When bacteria are individual cells in liquid, the rotation of their flagella generates a swimming motion. Conversely, bacteria, when immobilized on surfaces, form a biofilm, leading to higher resistance to antimicrobials and long-term colonization [40].

In our results, it should be noted that the total recovery rate of all bacteria was lower than 100%, suggesting that *S. Typhimurium* was strongly attached to the sand or the column and was starting biofilm formation. Furthermore, bacteria could reach crevices formed by the grains of sand via their flagella, leading to a better attachment [41] and to a lower recovery rate from the column. Similar results were obtained at a low flow rate by others [42]. They found that non-motile *E. coli* strains had higher retention time than motile *E. coli* strains in homogeneously packed sand column experiments. It showed that bacterial motility could be a factor in preventing bacterial attachment to solid surfaces. However, the BTCs of non-motile *Pseudomonas fluorescens* strains were found to be the first to emerge compared to motile bacteria at low water velocity [43]. Unfortunately, no data was available concerning the nature of the mutation present in the non-motile strain of this work. 

A lower average retardation factor for the Δ*flgKL* mutant was found compared to the Δ*motAB* mutant (Table 2). This may be due to the absence of flagella for Δ*flgKL*. It may have reduced the possibility of interaction with sandy grains, thus promoting the preferential transport of this strain through the porous media. The non-motile Δ*motAB* mutant has flagella, and they could be attached to the surface of the sand more easily. It is harder for the strain without flagella to be captured by the sandy grains. This result was in agreement with low retardation factors of Δ*flgKL*, indicating preferential transport and low retention in the porous media. Similar results were observed for *Azotobacter vinelandii* [44]. Flagellated strain had a higher deposition rate than a non-flagellated one on a quartz surface in a radial stagnation point flow cell setup [44]. Combined with the result of this study, it could be seen that flagella promote bacteria adsorption to different surfaces on different experimental scales, and flagellated bacteria have more probability of sticking on porous media.

WT had higher recovery from effluent than the Δ*motAB* mutant. WT had a more preferential transport in porous media than the Δ*motAB* mutant, which may explain the high mass recovery in the effluent observed. These results agree with the existing literature, suggesting a linear relationship between preferential transport and mass recovery in the effluent [10]. Due to its motility, WT is not only highly recovered in the effluent but also more retained in the sand compared to Δ*motAB* when it is in contact with the sandy grains (Table 2), in agreement with what has been reported in the literature. Thus, Zhang et al. (2021) [21] observed motile and non-motile *E. coli* Δ*fliC* strains and found that the motile strain was deposited more easily onto a quartz sand surface, grain-to-grain contacts, and narrow flow channels. It showed that the deposition of non-motile bacteria would be reduced in sand and water environments. The motile bacteria could be transported faster than bacteria without motility by providing a driving force for bacterial transport, which emphasized the importance of motility to bacteria during their movement in porous media. Different conclusions have been reported regarding the impact of cell motility on bacteria transport. Some authors suggested that the ability of the cell to swim is an important factor that enhances transport [45]. Others reported lower mass recovery of the motile strains compared to the non-motile ones. They suggested that small pores of the porous medium increase the possibility of the non-motile bacteria being trapped, causing a low recovery rate [10]. However, it should be noted that these results have been observed for different species: the motile *E. coli* and the non-flagellated *Klebsiella* sp., which renders it difficult for comparison, while in this work, different mutants of the same strain were investigated, giving a more accurate comparison between different experiments.

WT and Δ*motAB* mutants showed different behaviors in the observed RPs. The difference between the RPs of these two strains might be explained by bacterial near-surface swimming. When bacteria swim next to surfaces, it increases the possibility of being retained onto them. Therefore, *S. Typhimurium* flagellar motility is needed for reaching the host cell surface but is influenced by physical forces during “near-surface swimming” by increasing local bacterial density [46]. Furthermore, *E. coli* is known to have near-surface swimming on solid surfaces and to have three swimming modes: landing, near-surface swimming, and swimming away; the three processes require different dynamic conditions [47]. Thus, in this work, *S. Typhimurium* WT via near-surface swimming on the sand surface could keep an equilibrium among these three modes to maintain stable bacterial retention in each layer, while the non-motile strains are mostly captured by the sand surface of the first layer without flagella dynamic to swim away. Likewise, the Δ*motAB* mutant showed a delay in BTCs compared with WT. In this case, the non-motile flagella can add a physical constraint because they are most likely spreading to all directions around the bacterial body, as observed during the tumbling phase. Thus, the bacterial cells are occupying more space, leading to a potential increase in the residence time in the column.

The *fliC^ON^* mutant had faster transport in BTCs, higher recovery in the effluent, and a lower retention rate compared to the *fljB^ON^* strain. Furthermore, experiments performed by others have highlighted structural differences between *fljB^ON^* and *fliC^ON^* strains [48]. They found that FliC had a higher density in the D3 domain compared to FljB. This allows FljB to be more flexible and motile under high-viscosity conditions. Also, the *fljB^ON^* mutant has been found to stay longer because of near-surface swimming than the *fliC^ON^* mutant [24]. This observation can also explain why FliC promotes bacteria transport in porous media. It should be noted that the replicates BTCs of *fljB^ON^* showed better repeatability than *fliC^ON^*, probably reflecting the motility advantage of *fliC^ON^* compared to *fljB^ON^* described in previous studies. FliC flagella modulate the swimming behavior on the surface to facilitate the search of invasion sites which can be influenced by the column packing [24].

The Δ*fliB* mutant presented around half of the retention rate in the RPs in comparison to WT. It may probably be because the Δ*fliB* filament without methylation has lower hydrophobicity, causing lower attachment to sand. Hydrophobicity has been found to be a driving force in bacteria retention in porous media. Thus, a study involving *Campylobacter jejuni* and *E. coli* cells has demonstrated that the more hydrophobic *C. jejuni* had more attachment to porous media than *E. coli*. However, it should be noted that hydrophobicity’s impact on bacteria transport and deposition has mainly been studied between two very different bacterial species, making it difficult to reach an accurate conclusion [49]. Recently, the flagella methylation of *S. Typhimurium* has been found to adhere to hydrophobic surfaces of epithelial cells [16]. As observed in this work, when the hydrophobicity is high, the retention is higher.

The findings of this work could be useful to improve the risk assessment process of bacterial contamination in soil and groundwater. The pathogen concentrations and the weather conditions have already been used with hydrodynamic modeling to estimate the risk for public health [50,51,52]. Here, we observe that biological cell motility plays a clear role in the capacity of pathogens to reach groundwater. For that reason, we suggest that microbial motility should be studied on putative soil contaminants and added to risk assessment models. 

## 4. Materials and Methods

### 4.1. Bacterial Strains

**Figure 4:**
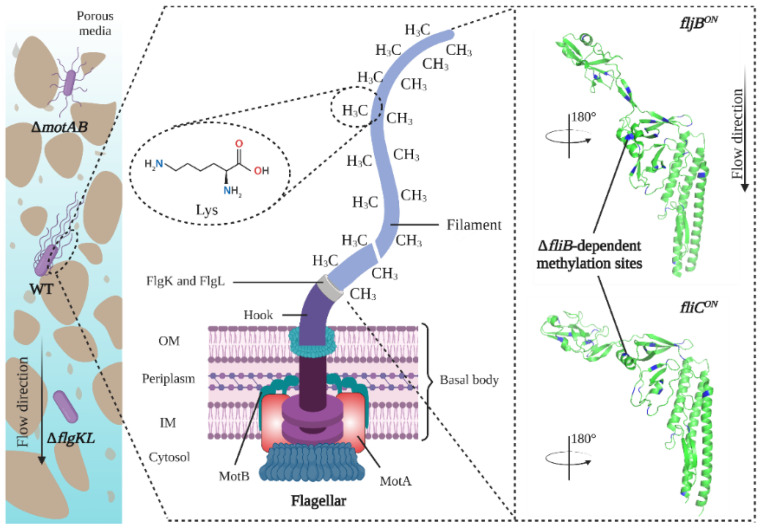
Schematic representation of the structure of bacterial flagellum.

The bacteria used in this study were all in *S. Typhimurium* SL1344 background and obtained as described here [16,24]. The different flagellar phenotypes used were: wild-type strain (WT), non-motile without flagella (Δ*flgKL*) [26], non-motile with flagella (Δ*motAB*) [36], motile with flagella unmethylated on lysine residues (Δ*fliB*), motile expressing only the flagellin FliC (*fliC^ON^*) and motile expressing one flagellin FljB (*fljB^ON^*) [16] (Table 1) FljB and FliC have different positions and orientations of flagellin in filament and FljB showed higher motility than bacteria expressing FliC under high viscosity [48]. *S. Typhimurium* have peritrichous flagella distributed all over the cells [36]. The structure of flagella includes the basal body, the hook, and the filament (Figure 4). 

Bacteria were inoculated on a lysogeny broth (LB) agar (35 g/L) Petri dish overnight at 37 °C, then bacteria strains were grown at 37 °C, 85 rpm overnight in LB (20 g/L). The growth curves of bacteria were measured by absorbance at OD_600_. Bacterial suspension harvested from the stationary phase of bacterial culture was used in this work. Batch experiments [53] were conducted to understand whether there was a change in bacterial concentration during column transport experiments in the absence of hydrodynamic flow conditions. Next, 250 mL conical flasks with 30 g Fontainebleau sand, 8 mL 0.1 mmol/L NaCl solution, and 2.5 mL bacteria suspensions were put on a shaker (85 rpm, 21 °C) for 45 min. The time for shaking was the same as the duration of the column transport experiments. The initial and final bacterial concentrations were determined by the number of colony-forming units (CFU) using the plating method [54] on LB agar. The result showed that there was not an obvious change in bacteria amount during the experiment. Accession numbers of FljB and FliC flagellin were PDB: 6 RGV and 1 IO1, respectively.

### 4.2. Column Transport Experiments

Transport experiments were performed in Plexiglass columns with an inner diameter of 3.4 cm and a length of 16 cm, packed with Fontainebleau sand. The particle size of Fontainebleau sand ranged from 0.16 to 0.79 mm, with a median diameter size of 0.36 mm. Two filter papers were used at the inlet and outlet of the column to make sure the solution came out without fine sand particles during the transport experiment. Before every experiment, the pump, tubes, and other column components were sterilized with bleach and 15 min of UV. Other materials were sterilized using an autoclave. The empty column with pipes, two caps, and two filter papers was weighted, and the mass was noted as *M*_0_ (g). The sand was inserted in successive layers into the column to assure homogenous distribution, and both column and sand were weighted and noted as *M*_1_ (g). The mass of sand was estimated as:

(1) $$M_{s}=M_{1}-M_{0}$$

and the sand bulk density (*ρ_b_*) was calculated by dividing the dry mass of the sand(*M_s_*) by the volume of the column (*V_c_*):

(2) $$\rho_{b}=M_{S}/V_{c}$$

The porosity (ε) was then estimated by the sand bulk density as follows:

(3) $$\varepsilon =\left(1-\rho_{b}/2.65\right)\times 100\%$$

The column was flushed upward using a background solution of NaCl (0.1 mmol/L), then the column was rinsed using the background solution reversed, and at the same time, the conductivity of the effluent solution was continuously measured at the column outlet until this parameter reached a stable value. Then, the column fully saturated with the background solution was weighed, and this mass was noted as *M*_2_ (g). Assuming that water density is 1 g/cm^3^, the volume occupied by water (*V_w_*) was estimated as:

(4) $$V_{w}=M_{2}-M_{1}$$

The saturation degree (*S*) could then be described as follows:

(5) $$S=V_{w}/V_{p}$$

here *V_p_* is the volume of the pores, calculated as

(6) $$V_{p}=V_{c}\times \varepsilon$$

**Figure 5:**
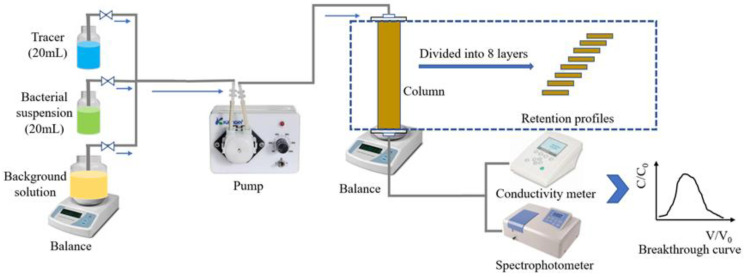
Schematic of column experiments.

For bacteria injection experiments, 20 mL of bacterial suspension was injected into each column at the initial concentration given in Table 2 for each replicate (Figure 5). The breakthrough curve of each strain was obtained by plotting bacterial concentration as a function of pore volume *V*/*V*_0_. Bacteria concentration was deduced by measuring the absorbance of effluent at OD_600_ at the column outlet. The bacterial suspension was flushed with a background solution (0.1 mmol/L NaCl solution) until the absorbance of the effluent returned to the baseline level. The ratio of bacteria mass recovered from the effluent (M_eff_) and retardation factor (R) was then calculated [55,56]. The retardation factor was calculated by the residence time of bacteria to the theoretical residence time of water in the column. M_eff_ corresponds to the ratio of the bacteria mass recovered at the column outlet to their mass injected at the column inlet. The retardation factor represents the ratio of the resident time of bacteria to the theoretical water resident time. 

After bacterial transport experiments, the sandy porous media inside each column was divided into 8 layers to explore the spatial distribution of the bacteria. First, a sterilized spoon was used to remove the sand into 8 flasks containing 0.1 mmol/L NaCl solution. The same solution was also used to wash the remaining sand off of the spoon. Then, we shook the flasks filled with sandy porous media for 15 min using an orbital shaker to isolate the retained bacteria from the sand. The Plating method count [54] was employed to determine the bacterial concentration in each layer of sand. The mass of bacteria recovered from all layers was regarded as M_retained,_ which was calculated by the total number of CFU retained in the sand divided by the CFU of the initial bacterial suspension injected into the column. The M_total_ is the sum of M_eff_ and M_retained_.

### 4.3. Simulation

The same modified mobile-immobile (MIM) model under two kinetic deposition sites, as described by Bai et al. (2016) [27], was used in this work. The two-region MIM model assumes that bacteria transport is limited to the mobile water region and that water in the immobile region is stagnant. Briefly, the one-dimensional bacterial transport model was described as [9]:

(7) $$\frac{\partial \theta C}{\partial t}+\rho \frac{\partial s_{1}}{\partial t}\;+\rho \frac{\partial s_{2}}{\partial t}=\frac{\partial }{\partial x}\left(\theta_{m}D\frac{\partial C}{\partial x}\right)-\frac{\partial qC}{\partial x}$$

where *θ* is the total water content, *C* is the bacterial concentration in the liquid phase (CFU/cm^3^), *t* is time (min), *ρ* is the bulk density of the porous media (g/cm^3^), *s*_1_ is the concentration of bacteria in the solid phase accounting for attachment or detachment, *s*_2_ is the concentration of bacteria in the solid phase related to irreversible straining, *x* is the distance (L), *θ_m_* is the mobile water content, *D* is the dispersion coefficient (cm^2^/min), and *q* is the Darcy velocity (cm/min). It was assumed that there was no bacterial exchange between the mobile and immobile regions. Then the bacterial mass transfer between solution and *s*_1_, *s*_2_ sites can be described as [57]:

(8) $$\rho \frac{\partial S}{\partial t}=\rho \frac{\partial \left(s_{1}+s_{2}\right)}{\partial t}=\theta_{m}\psi_{t}k_{att}C-k_{d}\rho s_{1}+\theta_{m}k_{str}\psi_{x}C$$

where *k_att_*, *k_d_*, *k_st_*_r_ are the first-order attachment, detachment, and straining coefficients, respectively (1/min); *ψ_t_* and *ψ_x_* describe the deposition of bacteria in the solid retention under time- and depth-dependent conditions, respectively. Another hydrodynamic parameter is *λ* (cm) which describes the dispersivity of the medium and can be estimated by [56]:

(9) $$\lambda =\frac{D_{m}*\theta_{m}}{q}$$

where *D_m_* is the dispersion coefficient of the mobile region (cm^2^/min). 

Transport (*θ_m_*, *λ*) and deposition parameters (*k_att_*, *k_d_*, *k_str_*) were obtained by fitting of theoretical MIM model, implemented in Hydrus 1D code (software version: Hydrus-1D_4.17, free in https://www.pc-progress.com/en/Default.aspx?Downloads (accessed on 7 June 2021)) to the experimental breakthrough curves of bacteria.

### 4.4. Statistical Analysis

To determine whether flagellar phenotypes affect bacterial transport and deposition in porous media, the replicates for each strain were analyzed with WT for significance using Student’s *t*-test.

## 5. Conclusions

In this study, the effects of *S. Typhimurium* flagellar phenotypes on bacterial transport and deposition in sandy porous media were explored. 

Flagella affected *S. Typhimurium* transport and deposition in porous media. *S. Typhimurium* strain with non-motile flagella reduced bacterial transport and increased bacterial retention in comparison to the strain without flagella. Flagella motility promotes bacterial transport, and this was confirmed by the higher retardation factor of the BTCs and the higher recovery of effluent of motile flagellated strains. As observed during host-pathogen interaction studies, flagella phases of *S. Typhimurium* have a different influence on bacterial movement in porous media. The *fliC^ON^* strain showed a faster transport and higher recovery in the effluent than the *fljB^ON^* strain, but the FljB flagellin promoted bacterial adhesion. Flagella methylation influenced *S. Typhimurium* transport and deposition in porous media, probably because of the higher hydrophobicity of the flagella filament’s outer surface.

Our results will lead to more robust transport models and could help to develop new environmental strategies through a more accurate risk assessment process. 

## Figures and Tables

**Table 1:** Cell properties for *S. Typhimurium* strains.

Flagellar Phenotypes	Wild Type	Δ*flgKL*	Δ*motAB*	*fljB^ON^*	*fliC^ON^*	Δ*fliB*
	EM774	EM4969	EM4939	EM1013	EM1012	EM3734
Cell motility	Motile	Non-motile	Non-motile	Motile	Motile	Motile
Flagella	On	Off	On	On	On	On

**Table 2:** Experimental conditions and mass balance information of *S. Typhimurium* transport experiments under saturated conditions.

Bacterial Strains	Replicate	Saturated Degree (%)	C Initial (CFU/mL)	Porosity (%)	Bulk Density (g/cm^3^)	Pulse Time (min)	Darcy Velocity (cm/min)	Retardation Factor	Recovery (%)		
									M_eff_	M_retained_	M_total_
WT	1	98.2	7.60 × 10^8^	37.88	1.65	2.40	0.906	0.94	51.97	34.64	86.61
	2	98.5	1.40 × 10^9^	36.41	1.69	2.33	0.936	0.79	46.49	26.99	73.48
	3	98.1	6.40 × 10^8^	37.98	1.64	2.37	0.939	0.86	42.83	27.35	70.18
								(0.86) ^a^	(47.1)	(29.66)	(76.76)
Δ*flgKL*	1	98.4	1.27 × 10^9^	38.92	1.62	2.20	1.010	0.66	44.41	17.69	62.10
	2	98.9	1.23 × 10^9^	38.26	1.64	1.85	1.191	0.66	44.83	22.41	67.24
	3	99.1	1.57 × 10^9^	37.91	1.65	2.23	0.991	0.65	44.87	29.69	74.56
								(0.66)	(44.7)	(23.26)	(67.97)
									0.42 ^b^	0.16	0.18
Δ*motAB*	1	96.4	1.30 × 10^9^	37.68	1.65	2.32	0.951	0.98	38.78	31.20	69.98
	2	97.9	1.73 × 10^9^	37.17	1.66	2.44	0.903	0.99	38.45	25.37	63.82
	3	96.6	1.93 × 10^9^	37.77	1.65	2.67	0.826	0.75	33.12	22.81	55.93
								(0.91)	(36.78)	(26.46)	(63.24)
									0.03	0.42	0.10
Δ*fliB*	1	98.4	9.00 × 10^8^	37.92	1.65	2.36	0.934	1.01	44.8	20.93	65.73
	2	97.3	1.60 × 10^9^	35.12	1.72	2.11	1.045	1.04	46.25	8.36	54.61
	3	99.5	9.33 × 10^8^	38.06	1.64	2.22	0.994	0.75	42.89	13.48	56.37
								(0.93)	(44.65)	(14.26)	(58.9)
									0.44	0.03	0.04
*fljB^ON^*	1	98.6	1.17 × 10^9^	36.84	1.67	2.07	1.066	0.79	40.44	33.58	74.02
	2	99.2	9.00 × 10^8^	36.79	1.67	2.34	0.941	0.70	38.34	17.76	56.10
	3	98.8	1.00 × 10^9^	36.91	1.67	2.36	0.935	0.84	38.98	14.46	53.44
								(0.77)	(39.25)	(21.93)	(61.18)
									0.04	0.29	0.13
*fliC^ON^*	1	93.5	1.57 × 10^9^	36.46	1.68	2.15	1.025	0.72	51.02	24.54	75.56
	2	96.0	1.03 × 10^9^	36.09	1.69	2.33	0.945	0.65	46.28	17.53	63.81
	3	100.0	1.10 × 10^9^	36.38	1.69	2.27	0.972	0.86	46.90	13.70	60.60
								(0.74)	(48.07)	(18.59)	(66.66)
									0.77	0.05	0.2

^a^ The values given were the mean value of the corresponding parameters, ^b^ the values given parallel were the *p*-values for analyzing significance with WT using *t*-test.

**Table 3:** Fitted parameters (replicates) for *S. Typhimurium* through saturated columns using the MIM model in HYDRUS-1D code.

Bacterial Strains	Replicate	*λ* (cm)	*θ_m_/θ*	*K_att_* (min^−1^)	*K_d_* (min^−1^)	*K_str_* (min^−1^)	*R^2^*
WT	1	0.69	0.54	3.12 × 10^−2^	3.93 × 10^−1^	7.87 × 10^−6^	0.9963
		(0.06) *	(0.004)	(1.58 × 10^−2^)	(2.10 × 10^−1^)	(6.56 × 10^−3^)	
	2	0.48	0.51	1.46 × 10^−1^	6.07 × 10^−1^	8.35 × 10^−2^	0.9958
		(0.07)	(0.008)	(1.88 × 10^−2^)	(6.09 × 10^−2^)	(1.25 × 10^−1^)	
	3	0.29	0.52	8.59 × 10^−2^	4.29 × 10^−1^	1.85 × 10^−1^	0.9963
		(0.03)	(0.002)	(1.36 × 10^−2^)	(8.16 × 10^−2^)	(2.49 × 10^−2^)	
Mean values		0.49	0.53	0.09	0.48	0.09	0.996
		(0.2001) **	0.0166	0.0574	0.1147	0.0929	0.0003
Δ*flgKL*	1	0.35	0.62	3.25 × 10^−2^	3.38 × 10^−1^	1.76 × 10^−1^	0.9987
		(0.03)	(0.002)	(5.00 × 10^−3^)	(1.17 × 10^−1^)	(6.23 × 10^−2^)	
	2	0.21	0.58	1.23 × 10^−1^	8.76 × 10^−1^	1.71 × 10^−1^	0.9968
		(0.04)	(0.004)	(1.82 × 10^−2^)	(1.71 × 10^−1^)	(8.46 × 10^−2^)	
	3	0.29	0.65	2.68 × 10^−2^	4.99 × 10^−1^	1.71 × 10^−1^	0.9948
		(0.05)	(0.003)	(2.52 × 10^−2^)	(9.80 × 10^−1^)	(6.95 × 10^−2^)	
Mean values		0.28	0.62	0.06	0.57	0.17	0.997
		0.0699	0.0375	0.0539	0.2761	0.0029	0.0020
Δ*motAB*	1	0.39	0.52	1.35 × 10^−1^	3.80 × 10^−1^	2.18 × 10^−1^	0.9793
		(0.14)	(0.03)	(3.95 × 10^−2^)	(7.83 × 10^−2^)	(3.14 × 10^−1^)	
	2	0.55	0.53	9.22 × 10^−2^	2.63 × 10^−1^	2.03 × 10^−1^	0.9668
		(0.20)	(0.04)	(4.15 × 10^−2^)	(1.37 × 10^−1^)	(4.68 × 10^−1^)	
	3	0.21	0.64	4.93 × 10^−2^	5.72 × 10^−1^	4.63 × 10^−1^	0.9969
		(0.02)	(0.002)	(5.48 × 10^−3^)	(1.37 × 10^−1^)	(5.45 × 10^−2^)	
Mean values		0.38	0.56	0.09	0.40	0.29	0.981
		0.1703	0.0654	0.0428	0.1560	0.1459	0.0151
Δ*fliB*	1	0.14	0.67	3.37 × 10^−1^	5.63 × 10^−1^	1.03 × 10^−1^	0.9823
		(0.04)	(0.02)	(1.83 × 10^−2^)	(5.74 × 10^−2^)	(1.12 × 10^−1^)	
	2	0.30	0.51	1.36 × 10^−1^	5.74 × 10^−1^	5.82 × 10^−2^	0.9852
		(0.13)	(0.02)	(5.11 × 10^−2^)	(1.33 × 10^−1^)	(3.21 × 10^−1^)	
	3	0.34	0.56	7.50 × 10^−2^	4.42 × 10^−1^	2.00 × 10^−1^	0.9989
		(0.03)	(0.003)	(4.58 × 10^−3^)	(4.35 × 10^−2^)	(5.62 × 10^−2^)	
Mean values		0.26	0.58	0.18	0.53	0.12	0.989
		0.1042	0.0852	0.1368	0.0732	0.0726	0.0088
*fliC^ON^*	1	0.32	0.65	2.99 × 10^−2^	6.92 × 10^−1^	1.69 × 10^−4^	0.9945
		(0.06)	(0.005)	(3.91 × 10^−2^)	(9.65 × 10^−1^)	(4.69 × 10^−2^)	
	2	0.59	0.40	2.87 × 10^−1^	8.25 × 10^−1^	1.57 × 10^−1^	0.9958
		(0.16)	(0.008)	(1.09 × 10^−1^)	(1.78 × 10^−1^)	(3.92 × 10^−2^)	
	3	0.35	0.59	6.01 × 10^−2^	5.37 × 10^−1^	5.01 × 10^−2^	0.9988
		(0.02)	(0.002)	(1.17 × 10^−2^)	(1.06 × 10^−1^)	(1.47 × 10^−2^)	
Mean values		0.42	0.54	0.13	0.68	0.07	0.996
		0.1496	0.1295	0.1407	0.1441	0.0804	0.0022
*fljB^ON^*	1	0.22	0.51	1.64 × 10^−1^	7.61 × 10^−1^	3.60 × 10^−1^	0.9985
		(0.02)	(0.002)	(2.10 × 10^−2^)	(8.43 × 10^−2^)	(2.04 × 10^−2^)	
	2	0.76	0.54	1.14 × 10^−1^	5.29 × 10^−1^	3.86 × 10^−1^	0.9947
		(0.24)	(0.02)	(6.74 × 10^−2^)	(2.03 × 10^−1^)	(2.07 × 10^−1^)	
	3	0.33	0.59	7.71 × 10^−2^	6.00 × 10^−1^	2.68 × 10^−1^	0.9972
		(0.04)	(0.005)	(6.61 × 10^−3^)	(9.98 × 10^−2^)	(1.00 × 10^−1^)	
Mean values		0.44	0.54	0.12	0.63	0.34	0.997
		0.2885	0.0416	0.0436	0.1185	0.0621	0.0019

* The values given were the standard error coefficients (S.E.Coeff) obtained from HYDRUS-1D code, ** The values given parallel were the standard deviation.

## Data Availability

All supporting data of the study are available from the corresponding authors upon request.
